# Proteomic analysis of human frontal and temporal cortex using iTRAQ-based 2D LC-MS/MS

**DOI:** 10.1186/s41016-021-00241-5

**Published:** 2021-05-05

**Authors:** Long Xu, Haidan Sun, Yang Zhang, Zhengguang Guo, Xiaoping Xiao, Xin Zhou, Kun Hu, Wei Sun, Bo Wang, Weiming Liu

**Affiliations:** 1 Department of Neurosurgery, Beijing Tiantan Hospital, Capital Medical University, No. 119 West Road, South Fourth Ring Road, Beijing, 100070 China; 2 China National Clinical Research Center for Neurological Diseases (NCRC-ND), Beijing, 100070 China; 3 Core Facility of Instrument, School of Basic Medicine Chinese Academy of Medical Sciences, Institute of Basic Medicine Peking Union Medical College, 5 Dong Dan San Tiao, Beijing, 100005 China; 4 State Key Laboratory of Brain and Cognitive Science, Institute of Biophysics, Chinese Academy of Sciences, 15 Datun Road, Beijing, 100101 China

**Keywords:** Frontal cortex, Temporal cortex, iTRAQ, Proteomics, 2D-LC-MS/MS

## Abstract

**Background:**

The human brain is the most complex organ in the body, and it is important to have a better understanding of how the protein composition in the brain regions contributes to the pathogenesis of associated neurological disorders.

**Methods:**

In this study, a comparative analysis of the frontal and temporal cortex proteomes was conducted by isobaric tags of relative and absolute quantification (iTRAQ) labeling and two-dimensional liquid chromatography-tandem mass spectrometry (2D LC-MS/MS). Brain protein was taken from relatively normal tissue that could not be avoided of damage during emergent surgery of the TBI (traumatic brain injury) patients admitted in Beijing Tiantan Hospital from 2014 to 2017. Eight cases were included. Four frontal lobes and 4 temporal lobes proteome were analyzed and the proteins were quantitated. Gene Ontology (GO), Ingenuity Pathway Analysis (IPA), and Kyoto Encyclopedia of Genes and Genomes (KEGG) pathway analysis were used to analyze the biological function of identified proteins, unchanged proteins, and differentially expressed proteins (DEPs).

**Results:**

A total number of 2127 protein groups were identified in the frontal and temporal lobe proteomes. A total of 1709 proteins could be quantitated in both the frontal and temporal cortex. Among 90 DEPs, 14 proteins were screened highly expressed in the temporal cortex, including MAPT, SNCG, ATP5IF1, GAP43, HSPE1, STMN1, NDUFS6, LDHB, SNCB, NDUFA7, MRPS36, EPDR1, CISD1, and RALA. In addition, compared to proteins expressed in the frontal cortex, 14 proteins including EDC4, NIT2, VWF, ASTN1, TGM2, SSB, CLU, HBA1, STOM, CRP, LRG1, SAA2, S100A4, and VTN were a low expression in the temporal cortex. The biological process enrichment showed that unchanged proteins between the frontal and temporal cortex mainly take part in regulated exocytosis, axon guidance, and vesicle-mediated transport. The KEGG pathway analysis showed that unchanged proteins between the frontal and temporal cortex mainly take part in oxidative phosphorylation, carbon metabolism, Huntington’s disease, and Parkinson’s disease.

**Conclusions:**

The majority of proteins are unchanged between the frontal and temporal cortex, and unchanged proteins are closely related to its function. Among DEPs, MATP (tau) is upregulated in the temporal cortex, closely related to Alzheimer’s disease (AD), and is one of the targets for the treatment of AD. CLU is downregulated in the temporal cortex which functions as an extracellular chaperone that prevents aggregation of non-native proteins. It was suggested that the temporal lobe may not be the “functional dumb area” of the traditional view, but could be involved in important neural metabolic circuits.

**Supplementary Information:**

The online version contains supplementary material available at 10.1186/s41016-021-00241-5.

## Background

The brain is one of the most complex organs in the body, makes up the largest portion of the central nervous system, and has the ability to affect most activities in the body. It plays an essential role in the emotional perceptions of events, memory processes, psychological processes, and linguistic and behavioral aspects [1]. Proteomics focuses on the large-scale global analysis of protein levels, functions, and interactions. It has been widely applied in basic studies and is promising in clinical research, in the areas of disease diagnosis and prognosis. However, proteomics of the brain remains challenging, as the brain contains multiple regions with different functions.

Though the extreme complexity of the human brain and the diverse cell types present increased difficulty, the field of neuro-proteomics is rapidly developing and has opened a new avenue in biomarker discovery in recent years, since the first report of the application of proteomics on brain samples in the 1990s [2]. The Human Brain Proteome Project aimed to establish a large-scale analysis of brains. Cerebrospinal fluid (CSF) was extensively profiled as it is the most widely used body fluid for neurological diseases. Recently, more than 3000 non-redundant proteins were identified in the normal CSF proteome [3]. Multiple reliable biomarkers of central nervous system disorders such as multiple sclerosis, Alzheimer’s disease, and Parkinson’s disease have been identified by MS-based proteomics methods [4].

Both the frontal lobe and temporal lobe are two of the four major lobes of the cerebral cortex in the brain. The frontal lobe, located at the front of the brain, covers a relatively large part of the human brain and plays an important role in problem-solving, working memory, storage, and executive processes [5]. The temporal lobe, located in the bottom middle part of the cortex, is involved in long-term memory, language recognition, and visual and auditory input [6]. Abnormalities of the frontal lobe are known to lead to schizophrenia, epilepsy, and some emotional disorders such as depression and bipolar disorder. Injury of the temporal lobe may also cause epilepsy, disorders of visual perception, altered personality, and affective behavior [7]. Proteomics technologies have been used to study the functions of these two lobes in previous studies. For example, disease-related alterations in the frontal cortex were reported in schizophrenia and major depressive disorder using a quantitative proteomic approach [8]. Oligodendrocyte and calcium homeostasis dysfunction in the anterior temporal lobe was identified as an important feature of schizophrenia using stable isotope labeling and shotgun proteomics [9].

The two lobes have some common functions, including congestive and emotional functions. The different protein expression patterns of the two parts of the cortex are pivotal to illustrate and understand the complex functions of the brain in response to a large variety of environmental stimuli and will further provide clues for disease diagnosis and prognosis. In the present study, we mainly focused on the in-depth identification of the two types of cortex by two-dimensional liquid chromatography-tandem mass spectrometry (2D-LC-MS/MS). We also aimed to identify differentially expressed proteins in the frontal and temporal lobe, in order to gain a better understanding of the functions of the two lobes, using iTRAQ labeling quantitation.

## Methods

### Ethics statement

The experiment was approved by the IRB (institutional review board) of Beijing Tiantan Hospital, and the approval No. is KY2014-021-02. All patients’ relatives signed informed consent.

### Instruments and reagents

A Triple-TOF 5600 mass spectrometer from AB sciex (Framingham, MA, USA) and an ACQUITY UPLC system (Waters, Milford, MA, USA) were used for the proteomic analysis.

HPLC-grade acetonitrile, formic acid, trifluoroacetic acid, and ammonium bicarbonate were purchased from Thermo Fisher (San Jose, CA, USA); iodoacetamide (IAM) and dithiothreitol (DTT) were purchased from Sigma (St. Louis, MO, USA). 8-plex iTRAQ labeling reagent was purchased from ABsciex (Framingham, MA, USA). Sequencing-grade trypsin was purchased from Promega (V5111, Madison, WI, USA).

### Experimental design

**Fig. 1:**
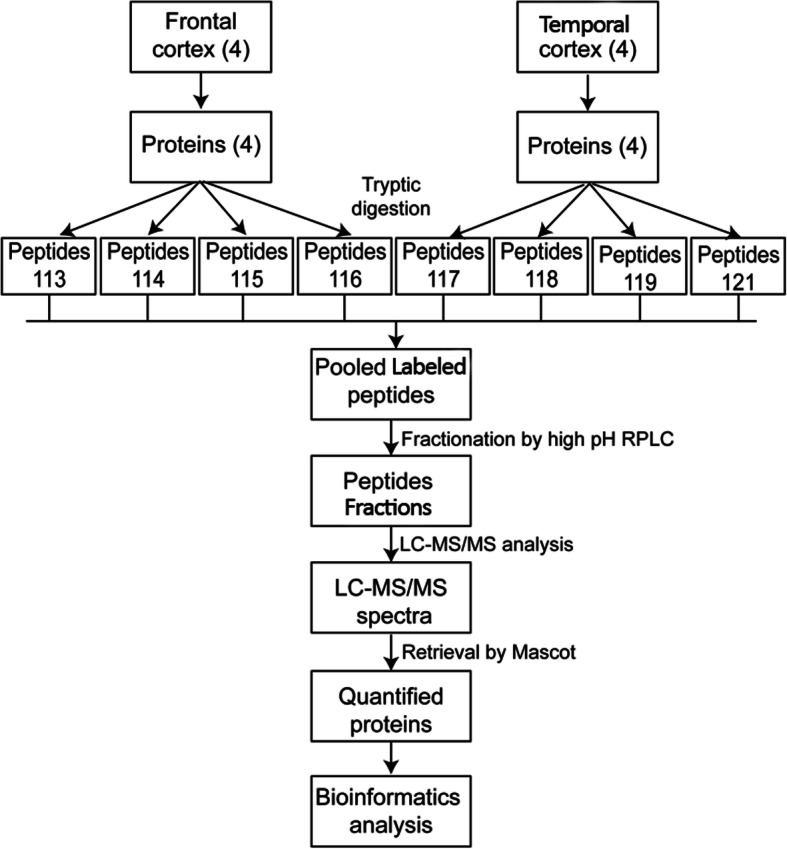
Workflow for proteomics analysis of frontal and temporal cortex

Brain protein was taken from relatively normal tissue that could not be avoided of damage during emergent surgery of the TBI (traumatic brain injury) patients admitted in Beijing Tiantan Hospital from 2014 to 2017. In this study, four frontal cortex and temporal cortex were used and the corresponding proteome was analyzed. A detailed scheme of experimental design for proteomics analysis of frontal cortex and temporal cortex is shown in Fig. 1. The main steps include (1) collection of the frontal cortex and temporal cortex; (2) extraction of total proteins from frontal cortex and temporal cortex; (3) protein reduction, alkylation, and tryptic digestion; (4) iTRAQ labeling of peptides from frontal cortex and temporal cortex; (5) combination of labeled peptides; (6) fractionation of combined peptides by high-pH RPLC (reversed-phase liquid chromatography); (7) LC-MS/MS analysis of fractionated peptides; (8) database retrieval of MS1 and MS2 spectra by Mascot for protein identification and quantification; and (9) bioinformatics analysis for functional annotations.

### Sample collection

Brain tissue samples, including four frontal cortex (Brodmann’s area 38) and four temporal cortex (Brodmann’s area 10), were obtained from 8 patients who underwent an operation for treatment of TBI patients (≤24 h) at the Beijing Tiantan Hospital of Capital Medical University (Beijing, China). The brain tissues sampled were relatively normal, more than 5mm away from the bleeding and contusion lesions, but resections were unavoidable during operations. Detailed information regarding these samples is listed in Table 1. These tissue samples were frozen immediately after resection and stored at −80 °C before use. The eight frozen tissue samples used for the proteomic analysis were first rinsed with PBS and homogenized by homogenizer in lysis buffer (50 mM Tris-HCl, 2.5 M thiourea, 8 M urea, 4% CHAPS, and 65 mM DTT) for protein extraction.

**Table 1:** Detailed information of all specimen in this study

	Frontal cortex 1	Frontal cortex 2	Frontal cortex 3	Frontal cortex 4	Temporal cortex 1	Temporal cortex 2	Temporal cortex 3	Temporal cortex 4
Age	51	45	42	57	41	58	43	44
Gender	Male	Male	Male	Male	Male	Male	Male	Male
Brodmann area	38	38	38	38	10	10	10	10
Diagnosis	Cerebral contusions and lacerations	Cerebral contusions and lacerations	Cerebral edema	Cerebral contusions and lacerations	Cerebral contusions and lacerations	Cerebral contusions and lacerations	Cerebral hemorrhage	Cerebral contusions and lacerations
Operation	Intracerebral hematoma clearance	Intracerebral hematoma clearance	Intracerebral hematoma clearance	Intracerebral hematoma clearance	Intracerebral hematoma clearance	Intracerebral hematoma clearance	Intracerebral hematoma clearance	Intracerebral hematoma clearance

### Protein reduction, alkylation, tryptic digestion, and iTRAQ labeling

The brain tissue proteins were digested using the filter-aided sample preparation (FASP) method [10] as described before. Briefly, proteins (200μg) were reduced in 20 mM DTT at 56 °C for 1 h and alkylated in 50 mM IAA in the dark for 45 min. Proteins were subsequently washed twice with 8 M urea and 50 mM ammonium bicarbonate. Finally, trypsin (enzyme: protein, 1:50, w/w) was added to each sample at 37 °C overnight. The digested peptides were then desalted using the Waters Oasis HLB column. The digested peptides (100μg) from 4 frontal cortex were labeled with iTRAQ reagent of 113, 114, 115, and 116 and the digested peptides (100μg) from 4 temporal cortex were labeled with iTRAQ reagent of 117, 118, 119, and 121 as 4 biological replicates, respectively, according to the manufacturer’s protocol. Moreover, 3 technical replicates were set up. Lastly, the labeled peptides were combined as one sample with 3 technical replicates. The combined samples were then dried by refrigerated centrifugal vacuum concentrator, desalted by HLB column, dried, and stored at −80 °C until fractionation by high pH RPLC.

### Fractionation of labeled peptides by RPLC

In order to decrease the complexity of labeled peptides mixtures and increase the identified depth of proteome, the labeled peptide mixtures were fractionated by a high-pH RPLC. Firstly, the labeled peptides mixtures (400μg) from frontal and temporal cortex were re-dissolved in 100μL 0.1% ammonium hydroxide solution (pH 10), then injected into an X-bridge Peptide BEH C18 column (4.6 mm×250 mm, C18, 3μm, 100 Å, Waters, MA, USA), fractionated with mobile phase A_1_ (0.1% NH_3_·H_2_O, pH=10) and mobile phase B_1_ (90%ACN/10%H_2_O/0.1%NH_3_·H_2_O, pH=10) at a 60-min gradient at flow rate of 1 mL/min. The eluted gradient was 5–30% buffer B_1_ for 60 min. The eluted peptides fractions were collected every minute and then concatenated into 20 fractions by combining fractions 1, 21, 41; 2, 22, 42, and so on. The 20 combined fractions were then dried by a refrigerated centrifugal vacuum concentrator and stored at −80 °C until analysis by LC-MS/MS.

### LC-MS/MS analysis

LC-MS/MS analysis of peptides in 20 fractions fractionated by high-pH RPLC from frontal and temporal cortex with 3 replicates was performed on an ACQUITY UPLC system coupled with Triple-TOF 5600 mass spectrometer (Framingham, MA, USA). Lyophilized peptides were re-dissolved in 0.1% FA and injected into a self-packed capillary LC column with reversed-phase C18 (75 μm×100 mm, 3μm) eluted with mobile phase A_2_ (0.1% formic acid) and mobile phase B_2_ (99.9% CAN/0.1% formic acid) at a 40-min gradient. The eluted gradient was 5–30% buffer B_2_ and at a flow rate of 300nL/min.

A Triple-TOF 5600 mass spectrometer was used to analyze the eluted peptides, and each fraction was run in triplicate. The mass spectrometer was operated in DDA mode. The MS data were acquired under high-sensitivity mode using the following parameters: full scan MS spectra (mass range from m/z 300 to 1800) were acquired in the TOF analyzer with a resolution of 40,000 at m/z 200: the top 30 precursor ions were selected from each MS full scan with an isolation width of m/z 2; they were fragmented in the CID collisional cell with rolling collision energy; Subsequently, MS/MS spectra were acquired in the TOF analyzer with a resolution of 20,000 at m/z 200; ions selected for MS/MS were dynamically excluded for a duration of 15s; the charge-state screening of precursor ions were set as 2–4 and a scan time of 100ms.

### Database retrieval of MS1 and MS2 spectra by Mascot for protein identification and quantification

All LC-MS/MS spectra were searched against the Swissprot database (taxonomy: homo sapiens, 20227 entries) by Mascot software (version 2.3.02, Matrix Science Ltd., London, UK). The search parameters were set as follows: the mass tolerance of parent and fragment ion was set to 0.05 Da; carbamidomethylating was set as the fixed modification; two missed cleavages were allowed. The search results were then filtered using Scaffold Q+ (version: 4.3.2) software. The false discovery rate (FDR) of retrieved peptide and protein were both set to equal or less than 1%, and each protein identification had to contain at least two unique peptides. Proteins that contained similar peptides and could not be differentiated based on MS/MS analysis alone were grouped to satisfy the principles of parsimony. The coefficients of variation (CV) of technical replicates and inter-individual were calculated as reported in previous studies [11].

### Statistical analysis

The Student test was used to screen differentially expressed proteins (DEP, *p*<0.05) between the frontal and temporal cortex. On the basis of *p*-value, fold change of equal or more than 1.3 means upregulated, and fold change of equal or less than 0.77 means downregulated.

### Bioinformatics analysis

All identified proteins were annotated by Gene Ontology (GO) analysis [12] with the Panther database (http://www.pantherdb.org/). Functional enrichment analysis of unchanged proteins and DEPs was carried out using the metascape website (https://metascape.org/gp/index.html#/main/step1) [13]. KEGG pathway analysis of unchanged proteins and DEPs was carried out using the DAVID website (https://david.ncifcrf.gov/) [14]. Ingenuity Pathway Analysis (IPA) software was used to find the related top canonical pathway and network.

## Results

In this study, brain tissue proteins from four frontal and four temporal cortex were collected. The detailed clinical information for 8 patients involved in this study is listed in Table 1. The proteomic profiling was performed based on brain tissue (Brodmann’s area 38 or 10). All patients were diagnosed with cerebral contusions and hemorrhage and underwent an operation of intracerebral hematoma clearance for treatment.

### Proteomics profile of frontal and temporal cortex

**Fig. 2:**
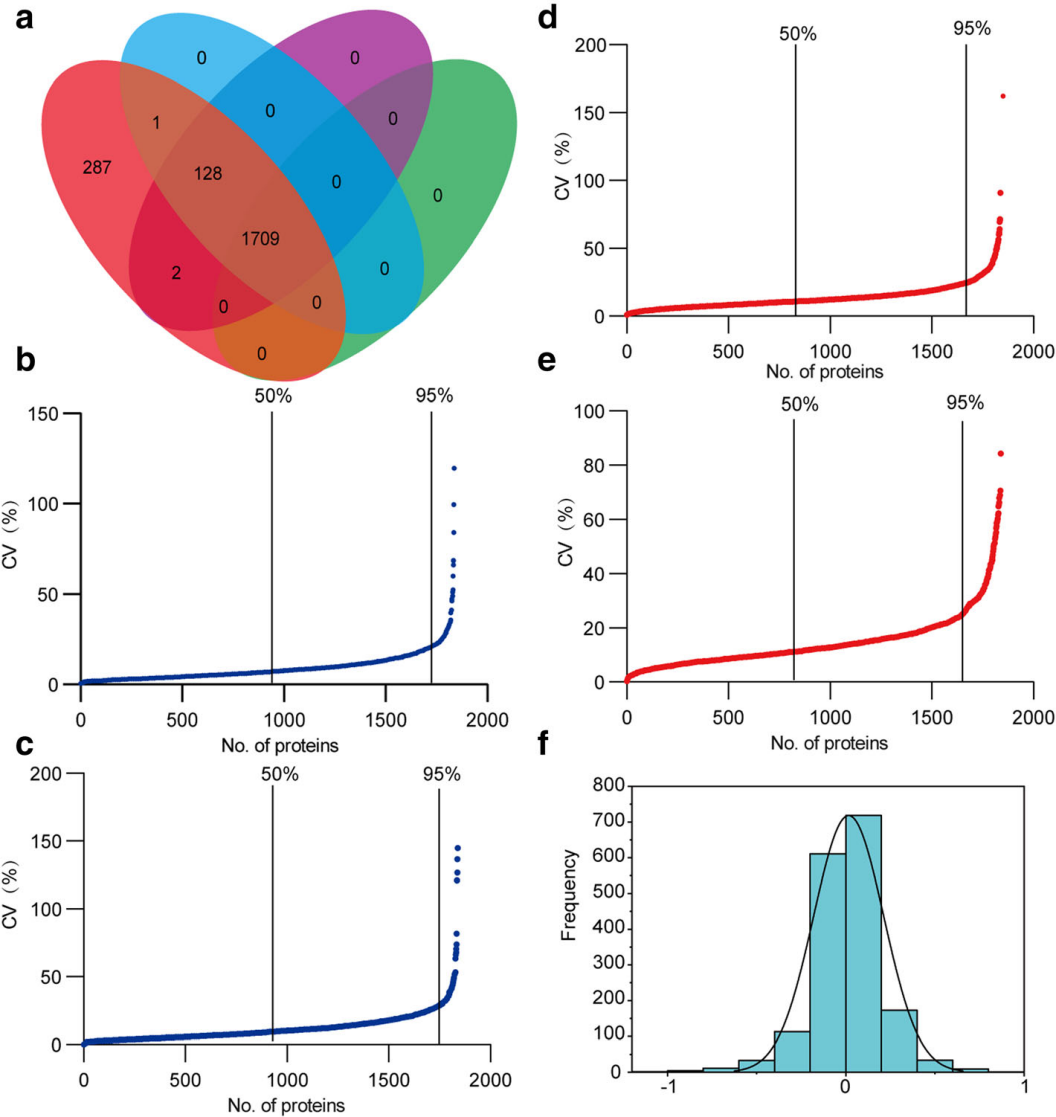
Proteome identified from frontal and temporal cortex and coefficient of variation of quantitative data. **a** Venn diagram of identified and quantified proteins, identified total proteins (pink ellipse, 2727), identified and quantified proteins from frontal cortex (blue ellipse, 1838), identified and quantified proteins from temporal cortex (purple ellipse, 1839), and quantifiable proteins both in frontal and temporal cortex (green ellipse, 1709). **b**, **c** Technical coefficient of variation of proteins identified from frontal and temporal cortex. **d**, **e** Inter-individual coefficient of variation of proteins identified from frontal and temporal cortex. **f** The distribution of quantified ratio between frontal and temporal cortex

In the experiment, a total number of 2127 protein groups were identified in either frontal or temporal cortex tissues (Fig. 2a). The protein group information is listed in Supplemental Table 1A. Of which, 1838 proteins could be quantitated in all four frontal cortex samples with three technical replicates and were used for technical variation analysis. The detailed protein information is listed in Supplemental Table 1B. The median of technical CVs was 6.95%, and 95% of the identifications (1746) showed a technical CV lower than 22.4% (Fig. 2b). In the temporal cortex proteome, a total of 2124 proteins were identified. Of which, 1839 proteins could be quantified (Fig. 2a). The information is shown in Supplemental Table 1C. The median technical CVs were 9.36%, and 95% of the identifications (1747) showed a technical CV lower than 28.8%, which was slightly higher than the CV in the frontal cortex proteome (Fig. 2c). In the following data analysis, the proteins with the highest 5% technical CVs were excluded to minimize the interference of technical variations. Finally, a total number of 1746/1747 proteins in frontal/temporal were used for an inter-individual analysis.

### Inter-individual variations in the frontal and temporal cortex proteome

In the frontal/temporal lobe proteome, the median inter-individual CVs from the analysis of 1746/1747 proteins were 11.3%/11.8%. The inter-individual CVs of 95% identifications in the frontal/temporal cortex samples were lower than 29.9/32.5 (Fig. 2d, e). The inter-individual CVs of the two brain tissue proteomes were similar to each other. Moreover, 1709 proteins were both identified and quantitated in the frontal and temporal lobe tissues (Fig. 2a). The detailed information is shown in Supplement Table 2A. The median inter-individual CVs were 11.3 and 11.8 respectively; the CVs of the two tissues were similar (Supplemental Figure 1A). Then the inter-individual CVs of 1709 frontal and temporal lobe proteins were plotted against each other. As shown in Supplemental Figure 1B, approximately most of the proteins had similar inter-individual variations between the two brain tissues. These results demonstrated that frontal and temporal lobe proteomes are both stable to some extent.

### Comparison of technical and individual variations

To view the correlation of technical and individual variations in the brain tissues, the CVs were plotted against each other. As shown in Supplemental Figure 2 A and B, proteins that demonstrated similar technical and inter-individual CVs were grouped close to the 45° line. In both frontal and temporal lobe tissues, most proteins exhibited larger inter-individual CV than a technical CV. Besides, compared with the median technical CVs 6.95%/9.36% observed between the different sequential runs, the median inter-individual CVs 11.3%/11.8% was larger. Those results indicated that the technical variation contributed less contribution to the total variation than the individual variation.

### Analysis of differentially expressed proteins

**Fig. 3:**
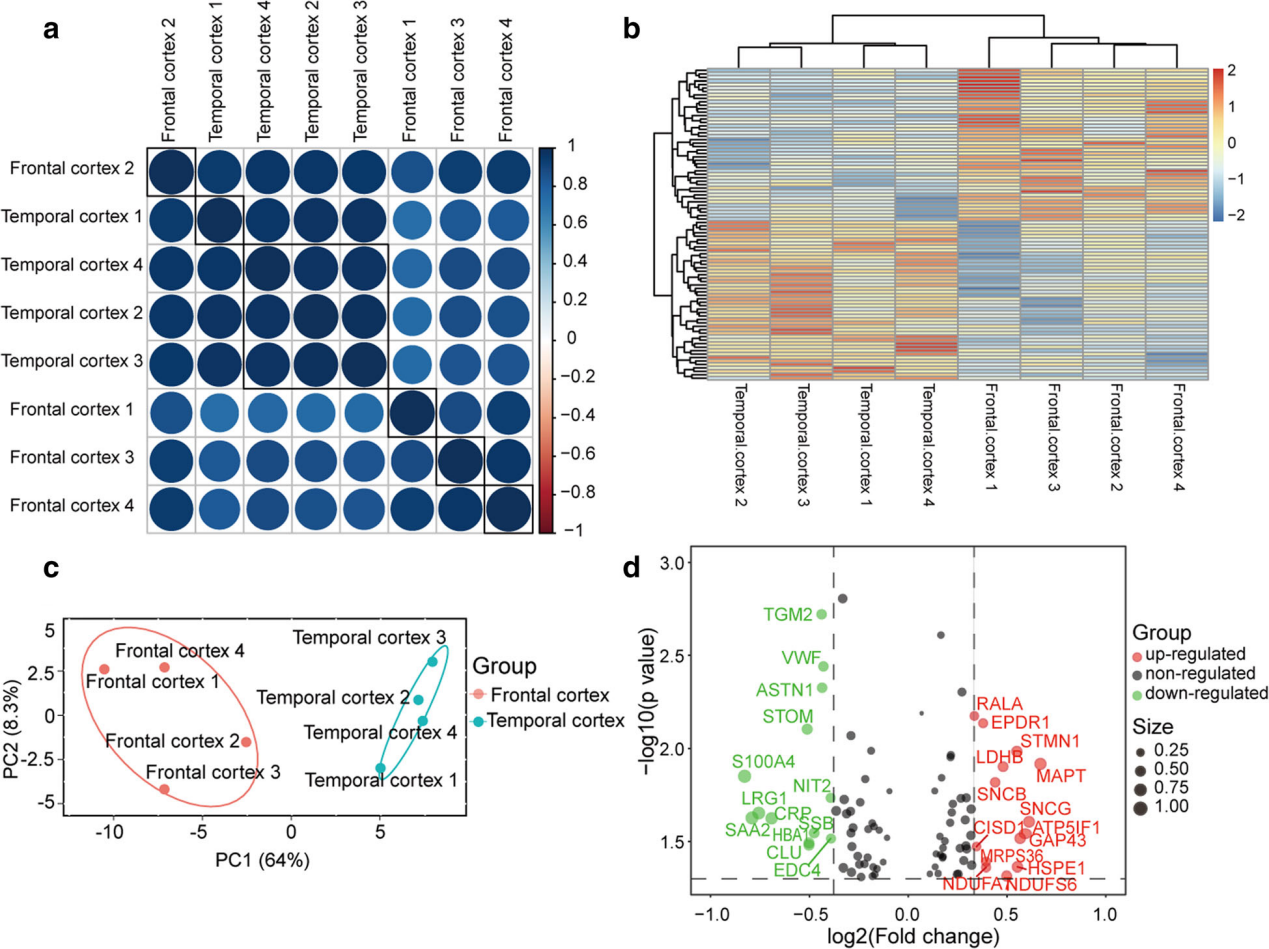
Differentially expressed proteins analysis of frontal and temporal cortex. **a** Correlation coefficient plot of differentially expressed proteins between frontal and temporal cortex. **b** Heatmap analysis of differentially expressed proteins. **c**, **d** Principal component analysis (PCA) and volcano plot analysis of differentially expressed proteins

**Fig. 4:**
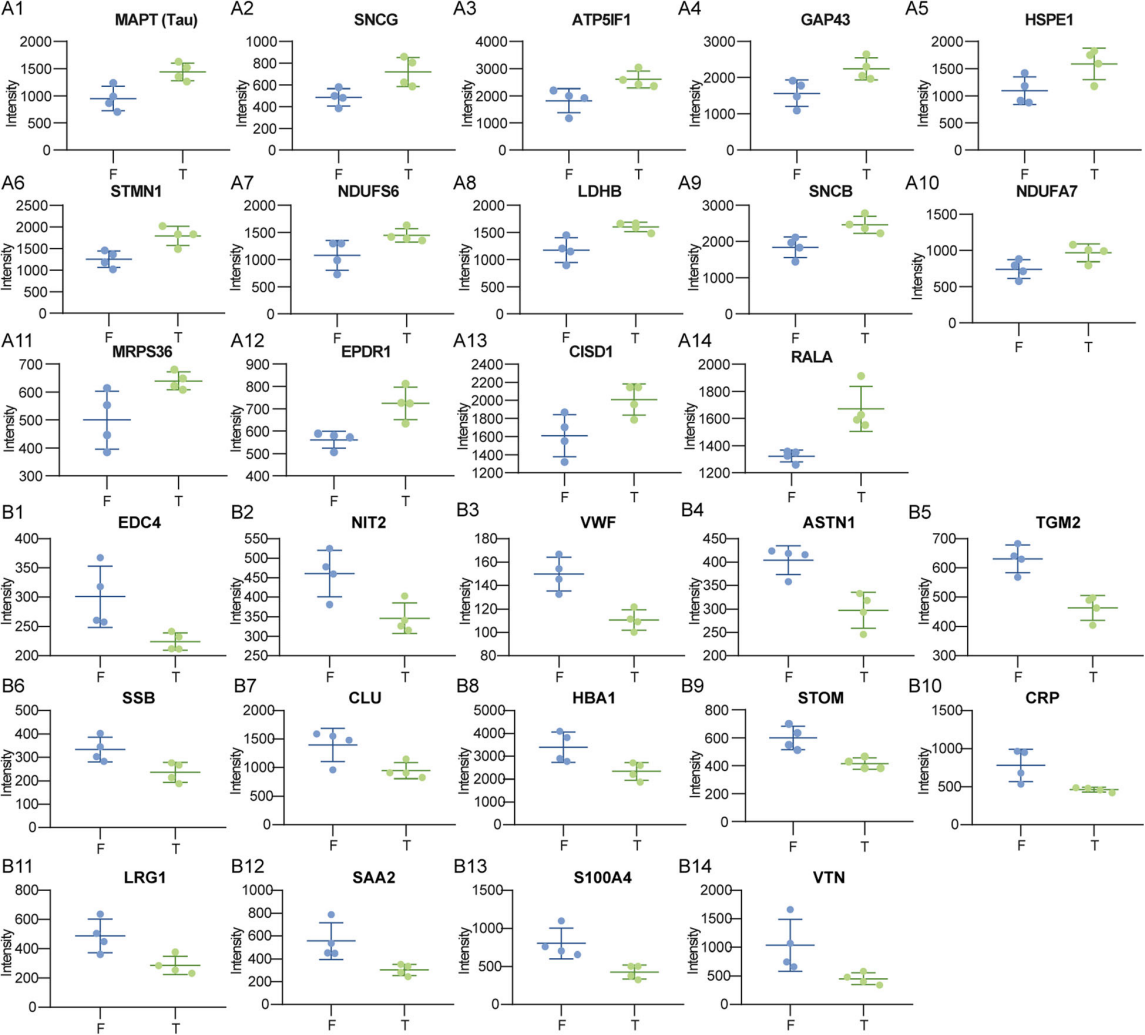
The expression of upregulated and downregulated proteins between frontal and temporal cortex. **A1**–**A14** The expression of upregulated proteins. **B1**–**B14** The expression of downregulated proteins

Differentially expressed proteins (DEPs) between the frontal cortex and temporal cortex were first assessed by Student’s test (*p*<0.05), and 90 DEPs are showed in Supplementary Table 2D. Correlation coefficient plot, heatmap, and principal component analysis (Fig. 3a–c) were used to investigate the dynamics and reproducibility of DEPs between frontal and temporal cortex. The frontal and temporal cortex can be differentiated into different clusters using PCA. Based on the *p*-value and 1.3-fold change, compared to proteins expressed in the frontal cortex, 14 proteins were screened highly expressed in the temporal cortex, including MAPT, SNCG, ATP5IF1, GAP43, HSPE1, STMN1, NDUFS6, LDHB, SNCB, NDUFA7, MRPS36, NDUFA, EPDR1, CISD1, and RALA. In addition, compared to proteins expressed in the frontal cortex, 14 proteins including EDC4, NIT2, VWF, ASTN1, TGM2, SSB, CLU, HBA1, STOM, CRP, LRG1, SAA2, S100A4, and VTN were a low expression in the temporal cortex (Fig. 3d). The expression pattern of these upregulated proteins in the temporal cortex are shown in Fig. 4A1–A14. The expression pattern of those downregulated proteins in the temporal cortex is shown in Fig. 4B1–B14.

### Bioinformatics and functional enrichment analysis

#### Functional annotation of frontal and temporal cortex proteins by Ingenuity Pathway Analysis (IPA)

**Fig. 5:**
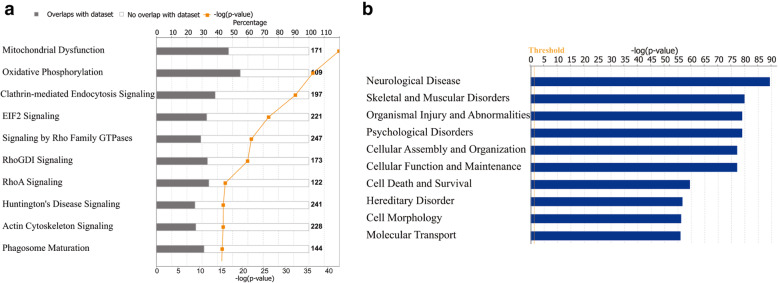
Pathway enriched with all quantified proteins in both frontal and temporal lobes. A total of 1709 proteins were quantified in the two brain regions. These proteins were mapped into canonical pathways using IPA. 95% confidence is indicated with the yellow threshold (**a**). Enriched disease and function by IPA annotation. A total of 1709 proteins which were both quantified in the two brain regions were mapped to IPA disease and function (**b**)

To further investigate the biological pathways, functions, and networks in the frontal and temporal tissues, we analyzed the identified proteins with the IPA software. The top ten significant IPA canonical pathways are listed in Fig. 5a. Among them, mitochondrial dysfunction was the most enriched pathway. Mitochondria are the primary consumers of oxygen in cells, responsible for ATP production and oxidative phosphorylation. Mitochondrial dysfunction may bring neurons a high risk of damage or death. In this experiment, numbers of identified proteins were categorized in mitochondrial dysfunction, which adds the evidence that brain function is importantly based on the normal function of mitochondria. Changes in brain proteins possibly lead to mitochondria dysfunction. The other pathways included oxidative phosphorylation, clathrin-mediated endocytosis signaling, and EIF2 signaling. Besides, when the physiological system development and function of these proteins were analyzed, the top function was predicated as nervous system development and function, and 532 of 1709 identified proteins were involved in this function. Moreover, when the disease and function annotations for the identified proteins were performed, the top results in a neurological disease (Fig. 5b), and 370 proteins were connected to this annotation. These results together show that proteins related to neurological function were enriched in brain tissue.

The frontal cortex, along with the other associational cortical areas, is involved in cognitive control processes. The temporal lobe is essential for visual memories and language comprehension. Not only do the protein constituents of the frontal and temporal cortex provide useful information regarding the physiology and pathology of the central nervous system, but they also offer valuable clues for neurological diseases. Many molecules were involved in the neurological diseases by IPA functional analysis (Supplemental Table 3). These diseases include Alzheimer’s disease, Huntington’s disease, and glioblastoma. According to IPA analysis, the top related disease was movement disorder, which had forty-seven categorized proteins. Most of these proteins are related to the inflammatory response, including CP, APOE5, and HP. For acute movement disorders in children, autoimmune and inflammatory disorders are the most common causes [15].

**Fig. 6:**
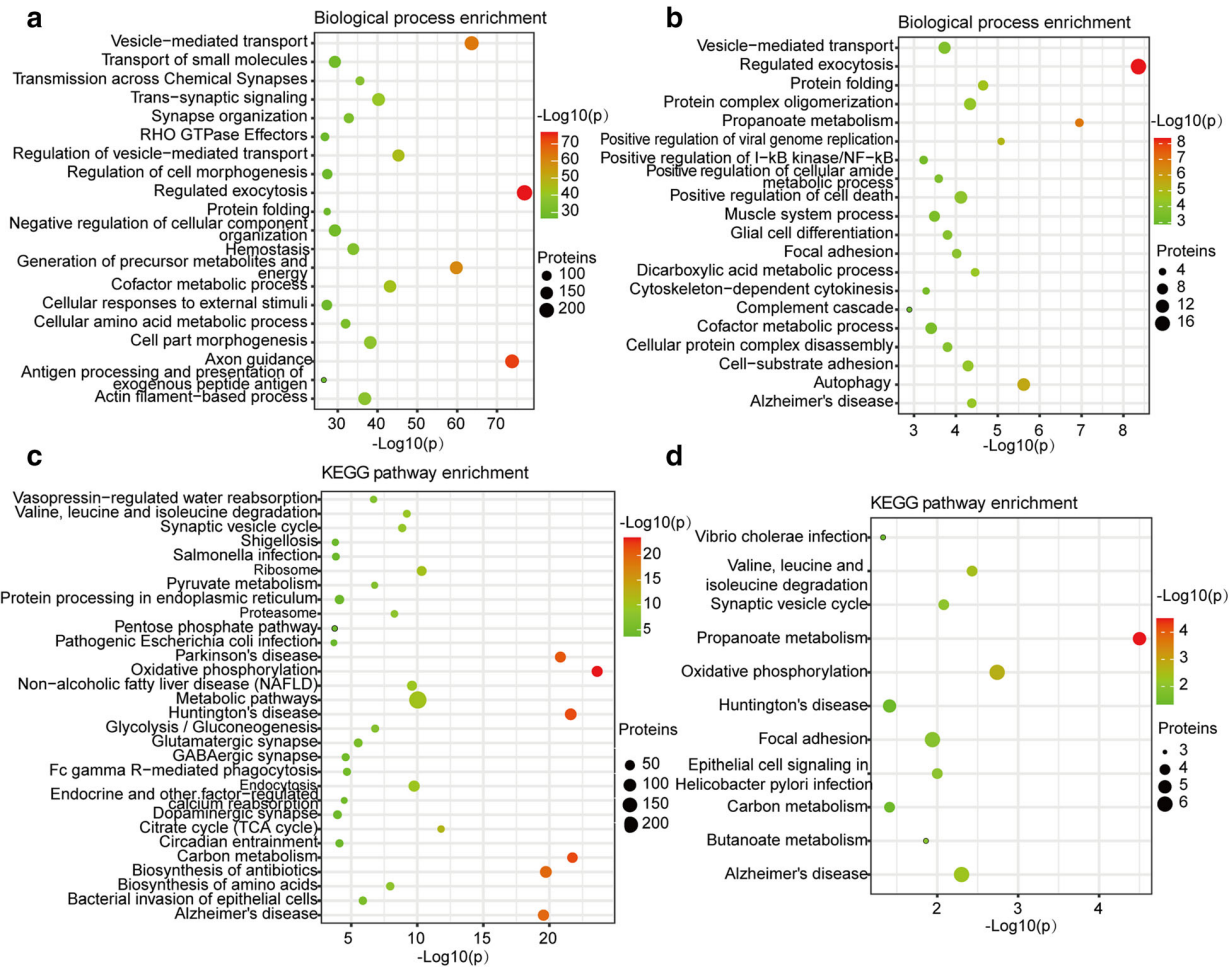
Functional enrichment analysis. **a** Biological process enrichment analysis of proteins without change between frontal and temporal cortex. **b** Biological process enrichment analysis of differentially expressed proteins between frontal and temporal cortex. **c** KEGG pathway analysis of proteins without change between frontal and temporal cortex. **d** KEGG pathway analysis of differentially expressed proteins in frontal and temporal cortex

Quantified proteins were separated into two parts based on *p*-value including 90 DEPs (*p*<0.05) and 1619 unchanged proteins (*p*≥0.05) between the frontal and temporal cortex. In order to further understand the functional differences between the two parts, functional enrichment analysis and KEGG signaling pathway analysis were carried out, respectively. As a result, the main biological processes enrichment of unchanged proteins are regulated exocytosis, axon guidance, vesicle-mediated transport, generation of precursor metabolites and energy, regulation of vesicle-mediated transport, cofactor metabolic process, trans-synapses, etc. (Fig. 6a, Supplemental Table 4A). The main biological processes enrichment of DEPs are regulated exocytosis, propanoate metabolism, autophagy, positive regulation of viral genome replication, protein folding, etc. (Fig. 6b, Supplemental Table 4B).

#### Pathway analysis of unchanged proteins and DEPs

To gain a deeper understanding of the pathway of unchanged proteins and DEPs between the frontal cortex and temporal cortex, we performed a KEGG pathway analysis.

The main KEGG signaling pathway of unchanged proteins are oxidative phosphorylation, Huntington’s disease, carbon metabolism, Parkinson’s disease, biosynthesis of antibiotics, Alzheimer’s disease, citrate cycle (TCA cycle), metabolic pathways, ribosome, endocytosis, non-alcoholic fatty liver disease (NAFLD), synaptic vesicle cycle, glutamatergic synapse, GABAergic synapse, dopaminergic synapse, etc. (Fig. 6c, Supplemental Table 4C). The main KEGG signaling pathways of DEPs are propanoate metabolism and oxidative phosphorylation (Fig. 6d, Supplemental Table 4D).

#### Functional analysis of part of upregulated and downregulated proteins in temporal cortex

Statistical analysis showed that compared to protein expression in the frontal cortex, a total number of 28 proteins were significantly upregulated or downregulated in the temporal cortex.

Microtubule-associated protein tau (MAPT, TAU) is one of the major microtubule-associated proteins in the vertebrate nervous system which promotes microtubule assembly and stability and might be involved in the establishment and maintenance of neuronal polarity [16]. Tau is specifically abundant in neurons of the central nervous system [17]. Tau is also closely associated with Alzheimer’s disease occurrence and progression [18]. Tau usually with multiple post-translational modifications (PTM) including phosphorylation, acetylation, disulfide bond, N-linked or O-linked glycosylation, isopeptide bond, methylation, and ubiquitination conjugation, and these PTMs as well as tau aggregation are closely related to its function and Alzheimer’s disease (AD). Tau protein is a component of the paired helical filaments associated with AD. Tau is also the main constituent of the intraneuronal neurofibrillary tangles (NFTs), which is one of the two main hallmarks of the disease in searching for disease-modifying therapies for AD [19]. Most importantly, tau protein is one of the targets and tau-targeting therapies for AD including educing tau expression, tau protein modifications inhibitors, tau aggregation inhibitors, and tau immunotherapies. Currently, the majority of tau-targeting therapies in clinical trials are immunotherapies, which have shown promise in numerous preclinical studies. Other approaches have been discontinued because of toxicity and/or lack of efficacy. Tau mRNA expression at the gene level had no obvious regional variability in a previous study [20]; however, in this study, a higher abundance of MAPT was found in the temporal cortex. As a result, the regional distribution of MAPT protein in the brain may be very important, especially for Alzheimer’s disease studies. From the results of this study, compared to the frontal cortex, tau protein is higher expressed in the temporal cortex.

Gamma-synuclein (SNCG) is a phosphoprotein highly expressed in the brain, particularly in the substantia nigra. SNCG plays a role in neurofilament network integrity. May be involved in modulating axonal architecture during development and in the adult.

Neuromodulin (GAP43) is an important component of the presynaptic terminal and axon. Alterations in cerebrospinal fluid levels of GAP43 may aid in the clinical diagnosis of frontotemporal dementia [21].

Stathmin-1 (STMN1) performs an important function in the regulation of microtubule dynamics and neurite elongation. For patients with intractable temporal lobe epilepsy, the expression of STMN1 was significantly decreased in the neuronal membrane and cytoplasm than in healthy controls [22].

The S100 proteins family are associated with anti-inflammatory processes and are always related to brain injury [23]. The protein S100A4 belongs to S100 proteins family was upregulated during brain injury and considered a therapeutic target in neuronal survival and neuroprotection via JAK/STAT and the IL-10 receptor [24]. Overexpression of S100A4 modulates varieties of antioxidant enzymes and neuroprotective genes. In this study, the S100A4 protein had significantly higher expression in the frontal lobe. In a previous study, the expression of S100A4 was also weak in the hippocampus and the temporal cortex in the human fetal brain [25].

Clusterin (CLU) functions as an extracellular chaperone that prevents aggregation of non-native proteins, prevents stress-induced aggregation of blood plasma protein s[26], and inhibits the formation of amyloid fibrils by APP, APOC2, B2M, CALCA, CSN3, SNCA, and aggregation-prone LYZ variants in vitro [27, 28]. CLU is an important element in the control of extracellular protein misfolding including Alzheimer’s beta-peptide.

C-reactive protein (CRP) displays several functions associated with host defense: it promotes agglutination, bacterial capsular swelling, phagocytosis, and complement fixation through its calcium-dependent binding to phosphorylcholine. The concentration of CRP in plasma increases greatly during the acute phase response to tissue injury, infection, or other inflammatory stimuli.

Astrotactin-1 (ASTN1) is located on the neuronal cell surface that mediates neuron migration [29]. These may help to understand the difference of the function in the two brain regions.

## Discussion

### Correlation of individual protein variations

In this study, the technical and inter-individual variations in frontal and temporal cortex proteomes were analyzed with iTRAQ based 2D-LC-MS/MS. The majority of the proteins expressed stabley among different individuals. In the frontal cortex, 84.8% (1481/1746) of the proteins show the inter-individual variation of less than 20%, 15.2% of the proteins with higher variation (>20%). In the temporal cortex proteomes, 82.6% (1441/1745) of the proteins exhibited the inter-individual variation of less than 20%, 17.4% (304/1745) of the proteins had higher variation (>20%).

### Biomarker application of frontal and temporal cortex proteins

Tau protein is high abundance in the temporal cortex and very target for the treatment of AD. CRP is high in abundance in the frontal cortex and the signal of acute phase inflammation implied the donors may be in a highly inflammatory state before conducting the operation of intracerebral hematoma clearance. Several proteins are related to the inflammatory response, including CRP, APOE5, and HP. For acute movement disorders in children, autoimmune and inflammatory disorders are the most common causes.

The biological process enrichment showed that unchanged proteins between the frontal and temporal cortex mainly take part in regulated exocytosis, axon guidance, and vesicle-mediated transport. The KEGG pathway analysis showed that unchanged proteins between the frontal and temporal cortex mainly take part in oxidative phosphorylation, carbon metabolism, Huntington’s disease, and Parkinson’s disease. The biological process enrichment showed that DEPs between frontal and temporal cortex mainly take part in regulated exocytosis, positive regulation of cell death, and autophagy. The KEGG pathway analysis showed that DEPs between frontal and temporal cortex mainly take part in propanoate metabolism, oxidative phosphorylation, and valine, leucine, and isoleucine degradation with few proteins.

Neurofibrillary tangle is one of AD’s main pathological changes. Interestingly, Tau and CLU are in relation to neurofibrillary tangle, Tau is upregulated in the temporal cortex, and CLU is downregulated in the temporal cortex. CLU functions as an extracellular chaperone that prevents aggregation of nonnative proteins. The results of a small number of samples in this study show that Tau and CLU are differentially expressed in different brain regions of the frontal and temporal lobes. It indicates that neurodegenerative diseases (such as AD, which may be related to these proteins) may be related to the unregulated protein expression between different brain regions.

An unavoidable limitation of our study is the lackness of normal controls with health normal population though we selected samples more than 5mm away from the bleeding and contusion lesions. It is expected that further researches will focus on the comparative study of the uninvolved tissue in patients with cerebral contusion with other brain diseases or the brain of normal healthy controls.

## Conclusion

In this study, a comparative analysis of frontal and temporal cortex protein constituents was conducted by iTRAQ-labeling 2D-LC-MS/MS. A total number of 1709 proteins were identified with at least two unique peptides. Ninety DEPs were screened out. The majority of proteins are unchanged between the frontal and temporal cortex, and unchanged proteins are closely related to its function. Among DEPs, MATP (tau) is upregulated in the temporal cortex, closely related to AD, and is one of the targets for the treatment of AD. CLU is downregulated in the temporal cortex which functions as an extracellular chaperone that prevents aggregation of nonnative proteins. This study showed that the expression characteristics of Tau and CLU proteins in the temporal lobe were different compared with those in the frontal lobe. It was suggested that the temporal lobe may not be the “functional dumb area” of the traditional view, but could be involved in important neural metabolic circuits.

## Supplementary Information

**Additional file 1: Supplemental Table 1**. Identified/quantified proteome, technical and inter-individual variation for frontal and temporal lobes of all identified proteins. A. Protein identification and quantification data for frontal and temporal lobe. B. Protein quantification information, the technical variation and inter-individual variation for frontal lobe. C. Protein quantification information, the technical variation and inter-individual variation for temporal lobe.

**Additional file 2: Supplemental Table 2**. Protein identification and quantification data in both frontal and temporal lobe. A. Protein identification and quantification data in both frontal and temporal lobe. B. Quantified proteins with calculation of p-value and fold change. C. Unchanged proteins. D. Differentially expressed proteins. E. up-regulated and down-regulated proteins.

**Additional file 3: Supplemental Figure 1**. Comparison of the inter-individual variation of frontal and temporal cortex proteomes. 1709 proteins which were both detected in frontal and temporal lobe proteins were analyzed. A. Distribution of inter-individual variations of frontal and temporal proteomes are shown. B. Scatter plots of the inter-individual variation for frontal and temporal proteins. The CV determined for the inter-individual variation of frontal is plotted against the inter-individual variation of temporal. The spots close to the 45°-line mean similar CVs of proteins in the two tissues.

**Additional file 4: Supplemental Figure 2**. Scatter plots of technical and inter-individual variations for frontal and temporal cortex proteins.

**Additional file 5: Supplemental Table 3**. Proteins involved in neurological disease. The function annotation, p value and molecules were displayed.

**Additional file 6: Supplemental Table 4**. Functional enrichment analysis of unchanged proteins and DEPs between frontal and temporal cortex in metascape and DAVID website. A. Biological process enrichment of unchanged proteins. B. Biological process enrichment of DEPs. C. KEGG signaling pathway enrichment of unchanged proteins. D. KEGG signaling pathway enrichment of DEPs.

## Data Availability

The datasets used and analyzed during the current study are available from the corresponding author on reasonable request.

## Abbreviations

iTRAQ: Isobaric tags of relative and absolute quantification
2D LC-MS/MS: Two-dimensional liquid chromatography-tandem mass spectrometry
TBI: Traumatic brain injury
GO: Gene Ontology
IPA: Ingenuity Pathway Analysis
KEGG: Kyoto Encyclopedia of Genes and Genomes
DEPs: Differentially expressed proteins
MAPT: Microtubule-associated protein
SNCG: Gamma-synuclein
ATP5IF1: ATPase inhibitor, mitochondrial
GAP43: Neuromodulin
HSPE1: 10kDa heat shock protein, mitochondrial
STMN1: Cluster of stathmin
NDUFS6: NADH dehydrogenase [ubiquinone] iron-sulfur protein 6, mitochondrial
LDHB: Cluster of L-lactate dehydrogenase B chain
SNCB: Beta-synuclein
NDUFA7: NADH dehydrogenase [ubiquinone] 1 alpha subcomplex subunit 7
MRPS36: 28S ribosomal protein S36, mitochondrial
EPDR1: Mammalian ependymin-related protein 1
CISD1: CDGSH iron-sulfur domain-containing protein 1
RALA: Ras-related protein Ral-A
EDC4: Enhancer of mRNA-decapping protein 4
NIT2: Omega-amidase NIT2
VWF: von Willebrand factor
ASTN1: Astrotactin-1
TGM2: Protein-glutamine gamma-glutamyl transferase 2
SSB: Lupus La protein
CLU: Clusterin
HBA1: Hemoglobin subunit alpha
STOM: Erythrocyte band 7 integral membrane protein
CRP: C-reactive protein
LRG1: Leucine-rich alpha-2-glycoprotein
SAA2: Serum amyloid A-2 protein
S100A4: Protein S100-A4
VTN: Vitronectin
AD: Alzheimer’s disease
CSF: Cerebrospinal fluid
IRB: Institutional review board
HPLC: High-performance liquid chromatography
IAM: Iodoacetamide
DTT: Dithiothreitol
RPLC: Reversed phase liquid chromatography
FASP: Filter-aided sample preparation
DDA: Data-dependent acquisition
CID: Collisional induced dissociation
CV: Coefficients of variation
PTM: Post-translational modification
NFT: Neurofibrillary tangle
